# Identification of miR-26a as a Target Gene of Bile Acid Receptor GPBAR-1/TGR5

**DOI:** 10.1371/journal.pone.0131294

**Published:** 2015-06-24

**Authors:** Xiaosong Chen, Haixia Xu, Lili Ding, Guiyu Lou, Yan Liu, Yalan Yao, Liangwan Chen, Wendong Huang, Xianghui Fu

**Affiliations:** 1 Department of Plastic Surgery, The Union Hospital of Fujian Medical University, 29 Xinquan Road, Fuzhou, Fujian, 350001, China; 2 Division of Endocrinology and Metabolism, State Key Laboratory of Biotherapy, West China Hospital, Sichuan University, and Collaborative Innovation Center of Biotherapy, Chengdu, Sichuan, 610041, China; 3 Department of Diabetes Complications and Metabolism, Diabetes & Metabolism Research Institute, Beckman Research Institute, City of Hope National Medical Center, 1500 Duarte Road, Duarte, CA, 91010, United States of America; 4 Central Laboratory, Henan People's Hospital and Zhengzhou University People's Hospital, No.7 Wei Wu Road, Zhengzhou, Henan, 450003, China; Nihon University School of Medicine, JAPAN

## Abstract

GPBAR1/TGR5 is a G protein–coupled receptor of bile acids. TGR5 is known to regulate the BA homeostasis and energy metabolism. Recent studies highlight an important role of TGR5 in alleviating obesity and improving glucose regulation, however, the mechanism of which is still unclear. Here we report that TGR5 is involved in mediating the anti-obesity and anti-hyperglycemia effect of a natural compound, oleanolic acid. By comparing the miRNA profiles between wild type and TGR5^-/-^ livers after OA treatment, we identified miR-26a as a novel downstream target gene of TGR5 activation. The expression of miR-26a in the liver was induced in a TGR5-dependent manner after feeding the mice with a bile acid diet. TGR5 activation strongly increased the expression of miR-26a in macrophages, including the Kupffer cells in the liver. We further demonstrated that JNK pathway was required for miR-26a induction by TGR5 activation. Interestingly, we located the TGR5-responsive DNA element to a proximal region of miR-26’s promoter, which was independent of the transcription of its host genes. These results unravel a new mechanism by which bile acid receptor TGR5 activates a miRNA gene expression.

## Introduction

Recent studies have highlighted bile acid (BA) signaling as a new therapeutic target for the treatment of metabolic disorders, including obesity and type 2 diabetes (T2D) [1]. Two major mediators of BA signaling have been identified: the nuclear Farnesoid X Receptor (FXR), and G protein-coupled bile acid receptor 1 (GPBAR-1) or TGR5 [2]. FXR is a transcription factor that can directly regulate gene expression. In contrast, TGR5 is a plasma membrane-bound BA receptor that initiates specific downstream signaling pathways, thereby changing gene expression indirectly. TGR5 has broad and varied levels of expression in different tissues [3–5]. Functionally, TGR5 regulates several important biological processes, including BA metabolism, gallbladder relaxation, energy expenditure and inflammatory modulation [6–11]. The roles of TGR5 in regulating obesity and diabetes have recently attracted great interest, however, the mechanism of which is still largely unclear.

Obesity and its major co-morbidity, T2D, have reached an alarming epidemic prevalence without an effective treatment available, thus highlighting an urgent medical need for new treatment strategies [12]. Recently, miRNAs have attracted considerable attention as potential new therapeutic targets for different human diseases, including obesity and diabetes [13,14]. MiR-26a is one of these miRNAs that play pleiotropic roles in many biological functions, including the metabolic regulations [15,16]. We recently show that miR-26a promotes pancreatic cell differentiation and prevents obesity-induced insulin resistance and excessive lipid synthesis in the liver [15, 17], which suggests that it has potential as a therapeutic target for improving metabolic disorders. However, the regulation of miR-26a expression is unclear and the development of effective methods to increase miR-26a expression remains challenging.

Here we show that TGR5 is the receptor that partially mediates the anti-obesity and anti-hyperglycemia effect of a natural compound, oleanolic acid (OA). TGR5 activation increases the expression of miR-26a in macrophages through a JNK-dependent pathway. This study thus provides a potential link between bile acid receptor TGR5 and miR-26a expression.

## Materials and Methods

### Reagents

BMS-345541, SP600125, PD98059, and H89 were purchased from Calbiochem (San Diego, CA). Oleanolic acid (OA), Lipofectamine 2000 and Lipofectamine LTX as well as other reagents for cell transfection were obtained as we described previously [18].

### Animal maintenance and treatments

C57BL/6 wild type (WT), c-Jun-N-terminal kinase 1 (JNK1) knockout and TGR5^-/-^ mice in C57BL/6 background were described previously [5, 18]. Animal studies were carried out in strict accordance with the recommendations in the Guide for the Care and Use of laboratory Animals of the National Institutes of Health. The animal protocol was approved by the City of Hope Institutional Animal Care and Use Committee (IACUC). For studies involving high fat diet (HFD) and OA feeding, mice (n = 10) of 8 weeks of age were fed with HFD as previously described [7] or the control diet (n = 5) for 8 weeks. The HFD-fed mice were then divided into two separate groups (5 mice/group). One group were kept feeding with HFD, Another group was switched to HFD+OA (100 mg/kg, mixed with HFD) for another 8 weeks. For studies involving cholic acid (CA) feeding, 3–4 mice of 8 weeks of age were fed with either 1% CA or the control diet for 3 days. All animals received humane care and all study protocols complied with the institution’s guidelines. When the experiments are terminated, animals will be under anesthesia and euthanized by CO_2_. All euthanasia procedures will follow the recommendation of the Panel on Euthanasia of the American Veterinary Medical Association.

### Histology

When animal feeding was terminated, all mice were euthanized. Liver tissues were fixed in 10% formalin, dehydrated and embedded in paraffin. Hematoxylin and eosin staining was performed for standard histological examination by Pathology Core at City of Hope.

### Isolation and cultivation of Kupffer cells and peritoneal macrophages

Isolation and cultivation of both Kupffer cells and Peritoneal macrophages were described in detail previously [19].

### Metabolic Measurements

Blood glucose levels were determined using a portable glucose meter (Abbot Laboratories). For glucose tolerance test (GTT), mice were fasted for 16 hours and then injected intraperitoneally with D-glucose (2 g/kg body weight). Blood glucose levels were then determined immediately before glucose injection or 15, 30, 60 and 120 minutes after injection.

### Real-Time Polymerase Chain Reaction

The RNAs preparation and quantification by real-time polymerase chain reaction (RT-PCR) were described in detail previously [18]. Primer locations for miR-26a used for gene analysis were shown in S1 Fig.

### Transfection

Transient transfection was performed in a 96-well plate. Briefly, RAW264.7 cells were transfected with plasmids pGL3-TK-miR-26a-Luc using Lipofectamine LTX reagent according to the manufacturer’s instructions. For mIR-26a promoter analysis, different deletions of miR-26a promoter DNA sequences were cloned into pGL3-TK-Luc plasmids. To normalize the transfection efficiency, cells were co-transfected with pGL3-TK plasmid. Cells were treated with either DMSO or OA (10 μM) respectively for 24 hours. Luciferase activity was assessed using the Dual-Luciferase Reporter Assay System (Promega, Madison, WI) according to the manufacturer’s instructions. At least three replicates of each transfection were performed.

### Statistical Analysis

Results of experiments are presented as mean ± SEM. Two-tailed Student's t test was used to determine the significant differences between data groups (* *P* < 0.05, **P < 0.01, *** *P* < 0.001) unless otherwise indicated.

## Results

### TGR5 partially mediated the effect of OA on obesity and glucose regulation

**Fig 1:**
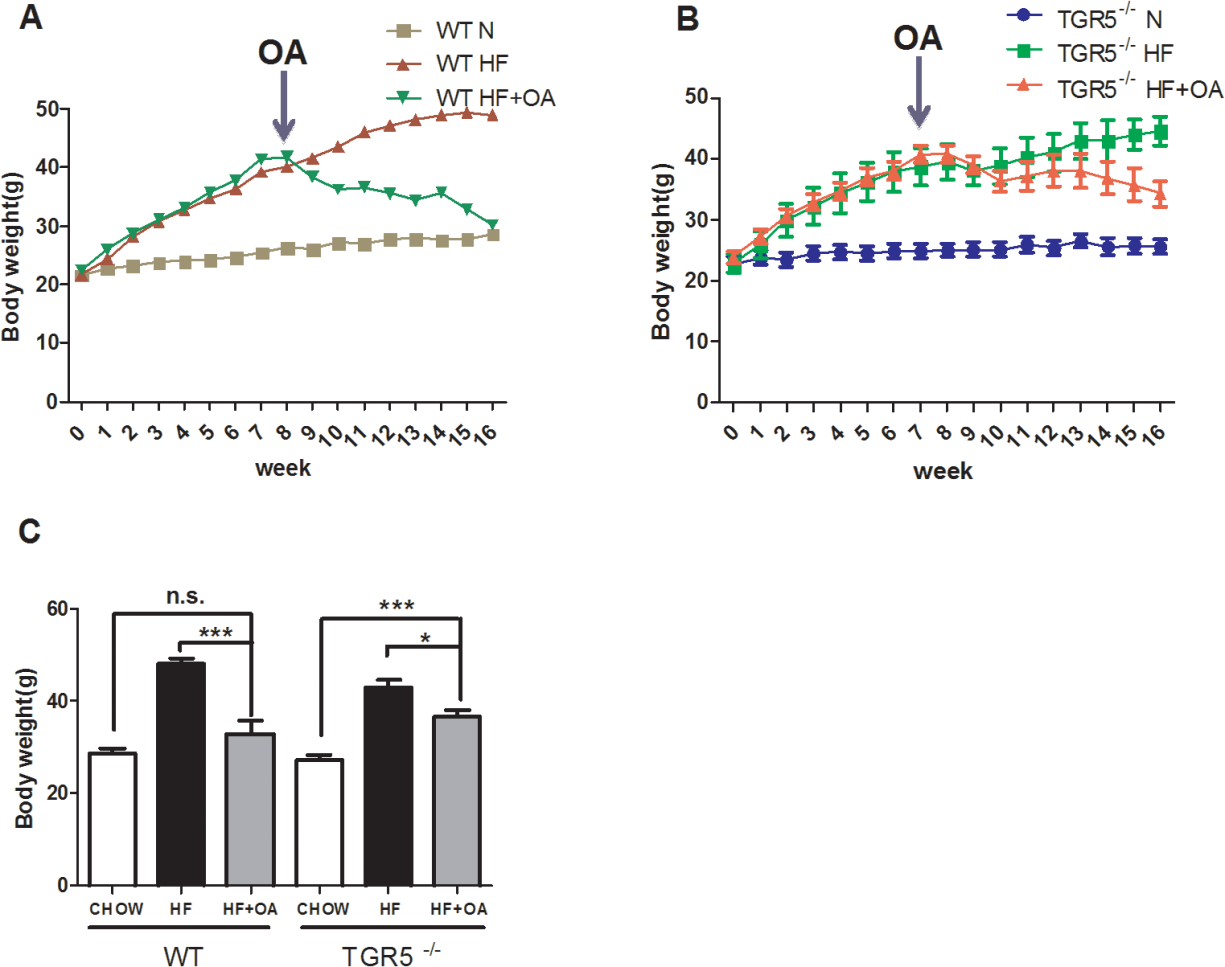
TGR5 mediated the suppressive effect of OA on obesity. (A, B) Changes of body weights of either WT or TGR5^-/-^ mice. WT (A) and TGR5^-/-^ mice (B) were fed a regular chow diet (N) or a HFD (HF). After 8 weeks, mice were divided into two groups randomly. One group was maintained the feeding with HFD, while the other group was fed HFD plus OA (100 mg/kg) (HF+OA). (C) Body weights of WT and TGR5^-/-^ mice when euthanized at 16^th^ week. Arrows indicate the starting point of OA treatment at 8^th^ week. N = 5, *p<0.05; ***p<0.01; n.s.: not significant.

**Fig 2:**
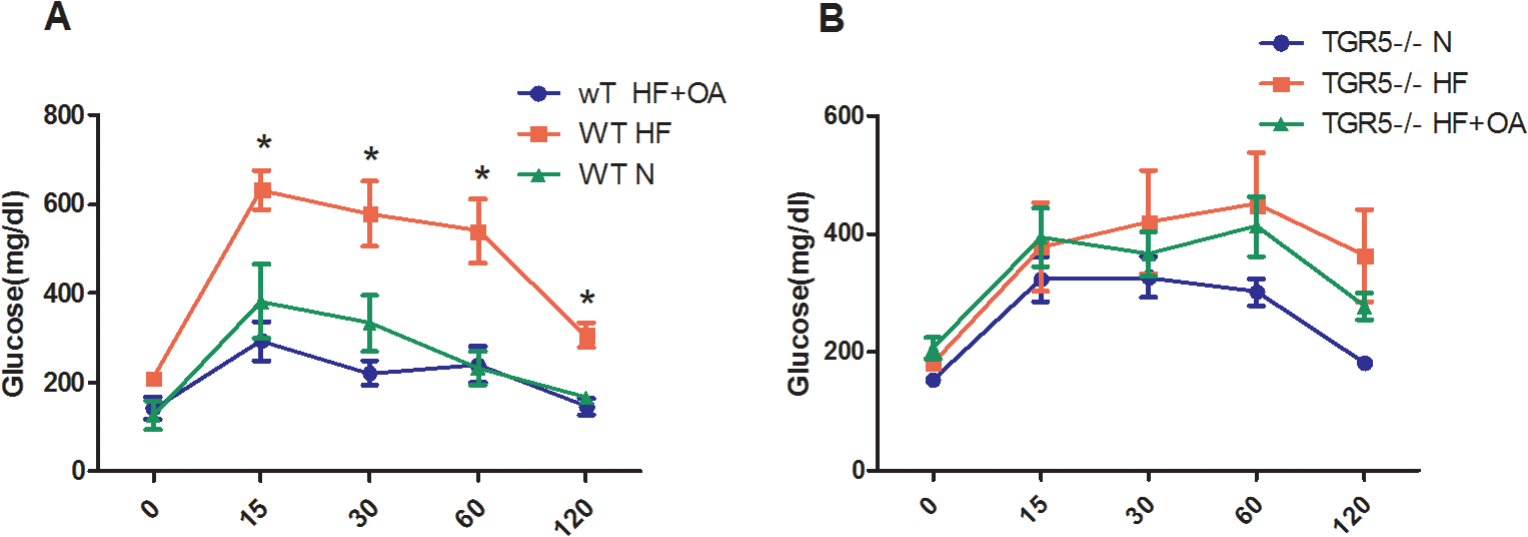
TGR5 mediated the effect of OA on improving glucose tolerance. (A, B) Glucose tolerance test on WT (A) and TGR5^-/-^ mice (B). The mice were treated as described in Fig 1. At the end of the feeding, mice were subjected to a glucose tolerance test. N: normal regular chow diet; HF: high fat diet; HF+OA: HFD+OA. * p<0.05.

OA is a triterpene isolated from olive leaves. It shows activities on TGR5 *in vitro* and displays effect on body weight control and anti-hyperglycemia in mice [20]. However, whether TGR5 is the receptor to mediate the metabolic effect of OA *in vivo* is unknown. Therefore, we first determined whether TGR5 was the mediator of OA’s beneficial effect in mice. Wild type (WT) and TGR5^-/-^ mice of 8 weeks of age were fed with HFD or the control regular chow diet. After 8 weeks, the mice fed with HFD were then randomly divided into two groups. One group were kept feeding with HFD. The other group was switched to HFD+OA for additional 8 weeks. Mouse body weights were monitored during the entire process. In both WT and TGR5^-/-^ mice, HFD significantly increased mouse BW. As expected, OA treatment starting at 8^th^ week significantly reduced the mouse BW compared to that of HFD control mice (Fig 1). In comparison, the anti-obesity effect of OA was significantly reduced in TGR5^-/-^ mice (Fig 1). At 16^th^ week of feeding, the experiment was terminated. The final BW were measured and compared. For WT mice, OA feeding significantly reduced HFD-induced BW to the level comparable to the chow diet control group. In contrast, although OA also significantly decreased the BW of HFD-fed group of TGR5^-/-^ mice, there was still significant difference between this group and chow diet-feeding group (Fig 1). To better demonstrate the effect of OA on body fat, we measured the weight of visceral fat and the results indicated a similar effect of OA on reducing the HFD-induced body fat accumulation in WT but not TGR5^-/-^ mice (S2 Fig). This result was further confirmed by the reduced fat accumulation in WT livers, but not in TGR5^-/-^ livers, after OA treatment (S3 Fig). Taken together, these results indicated that TGR5 was required to partially mediate the OA’s anti-obesity effect. The slightly remaining effect of OA on BW in TGR5^-/-^ mice could be due to the weak activities of OA on FXR or other proteins [21]. To determine the role of TGR5 in mediating the anti-hyperglycemia effect of OA, we performed and compared the glucose tolerance test (GTT) on WT and TGR5^-/-^ mice. As expected, HFD feeding resulted in glucose intolerance in both WT and TGR5^-/-^ mice. However, OA treatment significantly improved the glucose tolerance in WT (Fig 2) but not TGR5^-/-^ mice (Fig 2), indicating a TGR5-dependent effect of improving glucose regulation by OA treatment.

### TGR5 activation increased the expression of miR-26a

**Fig 3:**
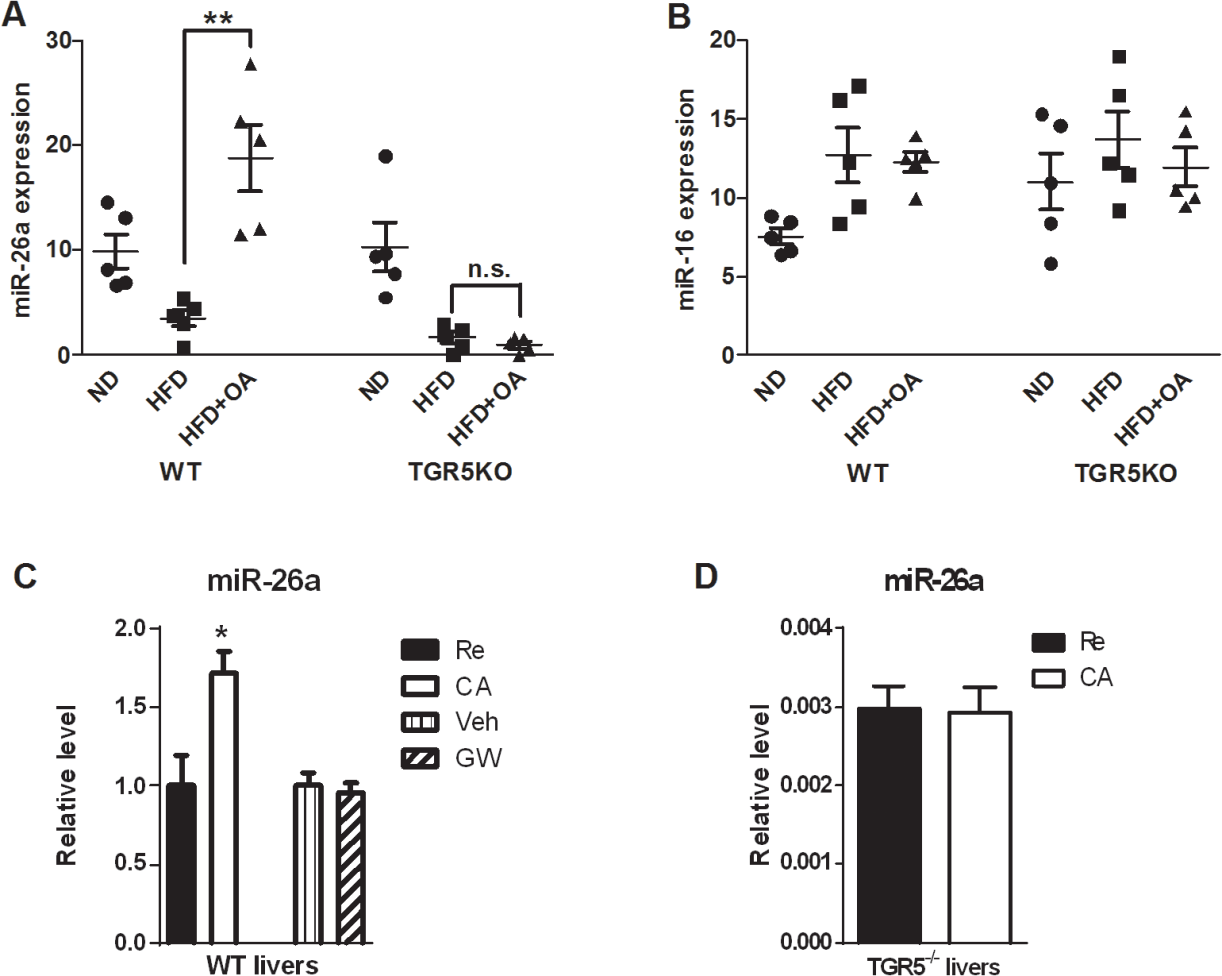
TGR5 activated the expression of miR-26a in the livers. (A, B) miR-26a (A), but not miR-126 (B), was significantly induced in WT but not TGR5^-/-^ livers after OA treatment. ** p<0.01. n.s.: not significant. ND: normal regular chow diet; HF: high fat diet; HF+OA: HFD+OA. (C) WT mice were fed with regular chow diet (Re) or 1% CA for 3 days. WT mice were orally gavaged with vehicle (Veh) or GW4064 (GW) once every day for 3 days. MiR26a expression in the liver was determined by QRT-PCR. (D) WT and TGR5^-/-^ mice were fed with regular chow diet (Re) or 1% CA. miR26a expression in the liver was determined by QRT-PCR. * p<0.05.

The above results indicated that OA was a TGR5 activator for its anti-obesity and anti-hyperglycemia effects. MiRNAs are recently demonstrated to participate in metabolic regulation and the effect of TGR5 activation on miRNAs has not been investigated. We therefore decided to compare the effect of OA treatment on miRNA expression in WT and TGR5^-/-^ mice. We screened for potential downstream liver miRNAs regulated by TGR5 activation, using the approach we have previously described [22]. Among the miRNAs we have searched, expression of miR-26a was strongly upregulated by OA treatment only in WT but not TGR5^-/-^ mice (Fig 3). In comparison, as a representative, expression of miR-16a was not affected by OA treatment or TGR5 status (Fig 3). Because BA signaling is the endogenous activator of TGR5, we then asked if miR-26a can be upregulated by BA signaling. We fed the mice with a 1% CA diet for 3 days as we described previously [18] and measured the expression levels of miR-26a. The results showed that CA feeding significantly upregulated miR-26a expression in the liver. Since CA feeding may potentially activate both TGR5 and FXR, we asked if FXR is involved in miR-26a regulation. We treated the WT mice with an FXR-specific agonist ligand, GW4064 compound, by oral gavaging. The results showed that FXR activation by GW 4064 had no effect on miR-26a expression (Fig 3 and S4 Fig). To further determine the role of TGR5 in mediating BA-induced miR-26a expression *in vivo*, we fed the WT or TGR5^-/-^ mice with 1% CA diet for 3 days and then measured the expression levels of miR-26a in the liver. The results showed that miR-26a expression in the liver was significantly increased after 1% CA diet feeding in WT but not TGR5^-/-^ mice (Fig 3). We did not observe the toxicity of CA to the mouse liver after three days feeding (data not shown). Similarly, treatment with a recently identified TGR5-specific ligand, 23(s)-m-LCA, strongly increased miR-26a expression in WT but not TGR5^-/-^ livers (S5 Fig)[23]. Taken together, these results clearly indicate a TGR5-dependent induction of miR-26a expression.

### TGR5 activation upregulated miR-26a expression in macrophages

**Fig 4:**
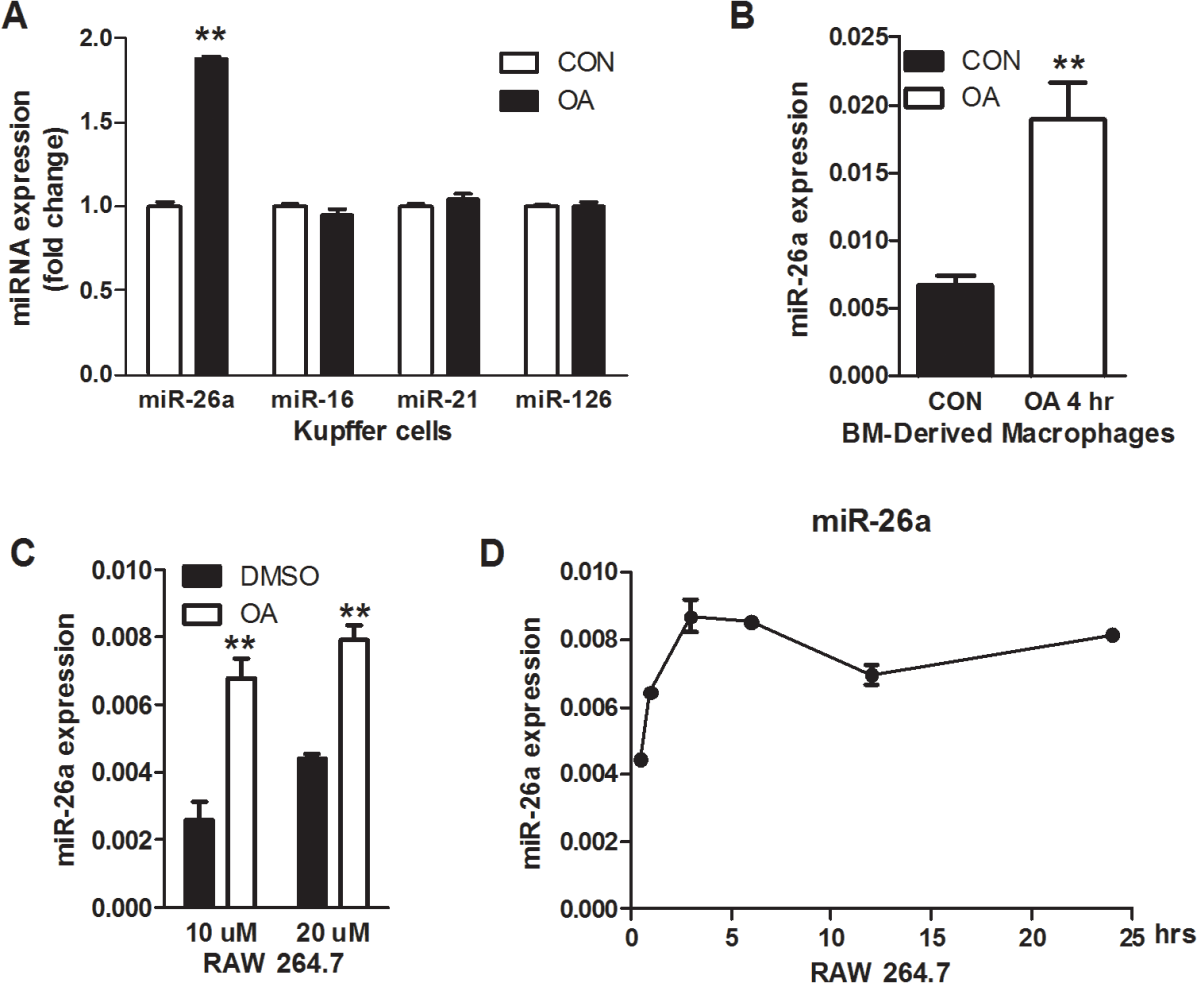
TGR5 activation induced miR-26a expression in macrophages. (A, B) Expression of indicated miRNAs in Kupffer cells (A) and bone marrow-derived macrophages (B) treated with OA (10 μM) for 24h. (C, D) Expression of miR-26a in RAW264.7 cells, a mouse macrophage cell line, treated with OA as indicated with different dose (C) or different time points (D) (OA, 10 μM). CON: vehicle control; OA: oleanolic acid. ** p<0.01.

In the liver, TGR5 is highly expressed in Kupffer cells, the liver resident macrophages. TGR5 is recently demonstrated to regulate macrophage migration [24]. We therefore asked whether TGR5 activation increased miR-26a expression in Kupffer cells. Treatment of Kupffer cells with OA (10 μM) resulted in strong increase of miR-26a expression levels compared to the control cells treated with vehicle (CON) (Fig 4). In contrast, other miRNAs, including miR-16, miR-21 and miR-126, were not affected by OA treatment in Kupffer cells (Fig 4). Similarly, upregulation of miR-26a expression was also observed in isolated bone marrow derived macrophages (BMDM) at as early as 4h by OA treatments (Fig 4). These results thus support a role of TGR5 in up-regulating miR-26a expression in macrophages. TGR5 is also expressed in a murine macrophage cell line RAW264.7 [18]. Therefore, we treated RAW 264.7 cells with OA and observed the same induction of mIR-26a expression in these cells (Fig 4). The induction dynamics curve showed a plateau of induction at about 3h by OA treatment (Fig 4). In comparison, other cell lines treated with OA had no effect on miR-26a expression (S6 Fig). We therefore use RAW264.7 cells as our cell model for the following studies.

### JNK pathway was downstream of TGR5 in miR-26a induction

**Fig 5:**
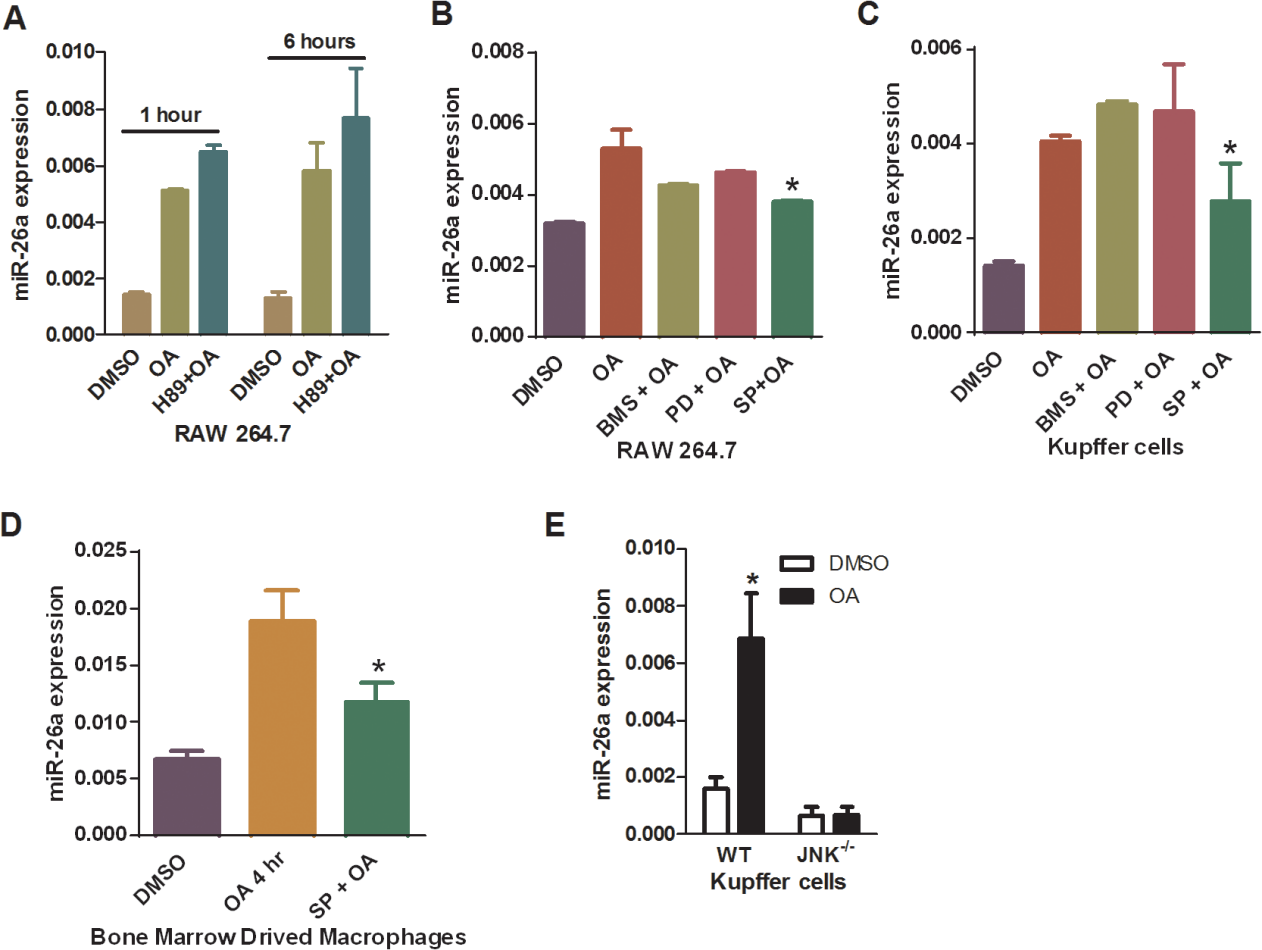
JNK pathway participated in TGR5-mediated miR-26a induction. (A-C) Expression of miR-26a in OA-treated RAW264.7 cells (A and B), Kupffer cells from WT mice(C), or BMDM from WT mice (D), which were pre-treated with or without indicated inhibitors against different signaling pathways. BMS: BMS-345541; SP: SP600125; PD: PD98059. (E) Kupffer cells were isolated from WT and JNK^-/-^ mice, respectively, and treated with OA or DMSO for 6 hours. MiR-26a levels were determined by QRT-PCR. *P<0.05.

Transducing signal through TGR5 results in intracellular cAMP generation and the subsequent activation of PKA [3]. Therefore, we asked if PKA was required for TGR5-dependent miR-26a induction. The results showed that OA-induced expression of miR-26a was not inhibited in RAW264.7 cells pretreated with a PKA inhibitor H89 (Fig 5), suggesting that PKA was not required for miR-26a induction by TGR5 activation. We then tested the effect of inhibitors of extracellular-regulated kinase (ERK), JNK as well as nuclear factor-κB (NF-κB) on miR-26a induction. Pre-treatment of RAW 264.7 cells with NF-κB inhibitor (BMS-345541), ERK inhibitor (PD98059) prior to TGR5 activation also did not affect the induction of miR26a by OA treatment (Fig 5). In contrast, JNK inhibitor SP600125 had significant effect to reduce the OA’s effect on miR-26a induction (Fig 5). Moreover, these results could be repeated in Kupffer cells (Fig 5) as well as in BMDM (Fig 5).

JNK is a member of mitogen activated protein kinases (MAPKs), which can be activated by TGR5 as we previously described [18]. Therefore, we further examined whether deletion of the JNK pathway could block the TGR5-dependent miR-26a induction. We isolated Kupffer cells from either wild type or JNK-/- mice and then subjected the cells to OA treatment. The results showed that miR-26a induction was increased in WT Kupffer cells but not in JNK^-/-^ Kupffer cells after OA treatment (Fig 5). These results clearly indicate that JNK was a critical downstream effector in TGR5-mediated miR-26a induction in Kupffer cells.

### TGR5 responsible DNA sequences were in the proximal regions of miR-26a promoter

**Fig 6:**
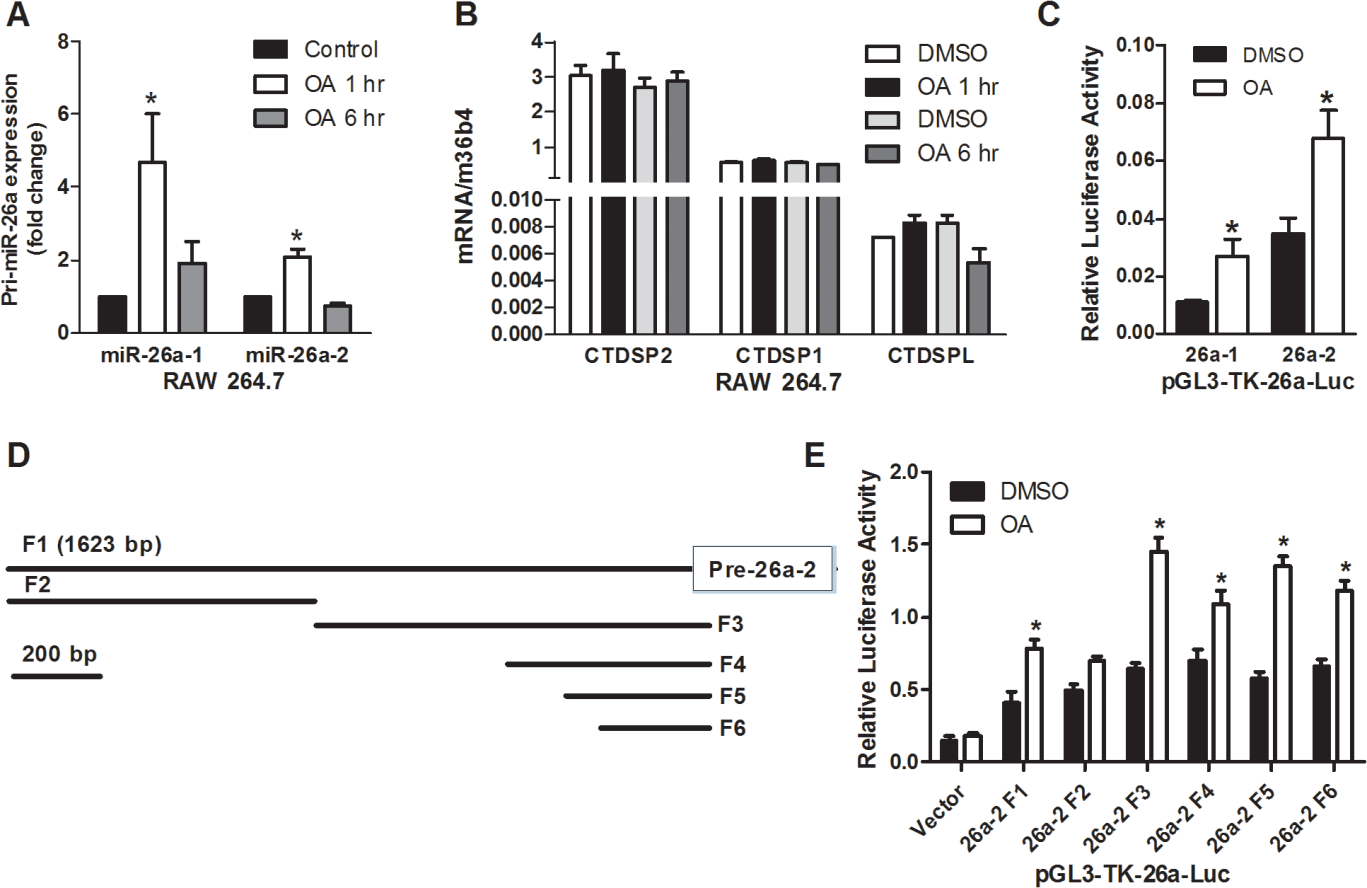
TGR5 responsible element was at the proximal regions of miR-26a promoter. (A) Induction of pri-miR-26a expression in RAW264.7 cells treated with OA (10 μM) for either 1h or 6h. (B) Expression of miR-26a host genes in RAW264.7 cells treated with OA (10 μM) for either 1h or 6h. (C) Relative luciferase activities in OA-treated (10 μM) RAW264.7 cells transfected with pGL3-miR-26a-1 and pGL3-miR-26a-2 vectors, respectively. (D) Schematic of upstream regulatory region of miR-26a-2 cloned into pGL3 luciferase vector. (E) Relative luciferase activities in OA-treated (10 μM) RAW264.7 cells transfected with pGL3 luciferase vector containing different miR-26a-2 regulatory DNA regions. *P<0.05.

The human *miR-26* family has two members, *miR-26a* and *miR-26b*. In both the human and mouse genomes, two loci align with *miR-26a*—*miR-26a-1* and *miR-26a-2*—which are located in intronic regions of *CTDSPL* and *CTDSP2*, respectively (S1 Fig). To better understand the mechanism by which TGR5 activation induced miR-26a expression, we compared the induction of miR-26a-1 and miR-26a-2 by OA treatment in RAW264.7 cells. The results showed that expression of both of pri-miR-26a could be significantly increased by OA treatment (Fig 6). However, when we measured the expression of miR-26a host genes: CTDSP2, CDTSP1 and CTDSPL, there was no effect of OA treatment on the expression of these host genes, indicating that expression of miR-26a was induced independent of its host genes and might have its own promoter in response to OA treatment (Fig 6). To test this possibility, we cloned the DNA fragments of promoters of miR-26a-1 and miR-26a-2 and generated luciferase reporters containing these promoters. Transfection assay in RAW264.7 cells indicated that both promoters responded to OA treatment (Fig 6). We then chose miR-26a-2 promoter for further analysis. After generation of luciferase reporters containing different deletions of miR-26a-2 promoter sequence (Fig 6), the shortest part of promoter containing about 200 bp proximal DNA sequences (26a-2 F6) still contained the element responsible to OA treatment (Fig 6). These results indicated that miR-26a might have its own promoter responsible to TGR5 activation and was under a distinct regulation from its host genes.

## Discussion

A paradigm shift in BA research is the concept expanding of BAs from mere surfactants to signaling molecules. Through activation of the two BA receptors, FXR and TGR5, BAs regulate their own metabolism as well as several other physiological processes. Functionally, both FXR and TGR5 response to BA signaling and they complement each other in several important BA signaling pathways, including BA homeostasis [25], liver regeneration [26,27], as well as obesity and T2D [2, 28]. On the other hand, these two BA receptors are different in their expression profiles, ligand selection and downstream regulatory pathways. Unlike FXR, which directly regulates the transcription of its target genes, activation of TGR5 generally triggers intracellular kinase pathways, which indirectly leads to changes of gene expression. In this study, we showed that the induction of miR-26a expression by either OA or BAs was dependent on TGR5. In contrast, FXR activation by its specific ligand had no effect on miR-26a expression. We also could not identify any FXR DNA binding elements in miR-26a promoter. Therefore, miR-26a could be a TGR5-specific target gene in response to BA signaling. Further studies will elucidate more differentiated functions of TGR5 and FXR in response to BA signaling.

Activation of TGR5 has been previously shown to inhibit LPS-induced expression of cytokines through increasing the intracellular cAMP levels and stimulating PKA activation [3]. However, our recent study demonstrates that activation of the TGR5 by its ligand in the absence of other pro-inflammatory stimuli such as LPS, leads to the up-regulation of IL-1β and TNF-α expression without PKA activation. Rather, the TGR5-dependent cytokine production is mediated by the activation of JNK [18]. Similarly, in activation of miR-26a expression, JNK, but not PKA, is required for the effect of TGR5 activation to increase miR-26a expression. These results indicate that TGR5 uses at least two different downstream signaling pathways for different biological functions. The differential activation of TGR5 downstream pathways under different conditions suggests that different TGR5 ligands may trigger different downstream pathways and gene expression, which leads to specific functional outcomes. As such, this should be taken into consideration during drug development of TGR5 ligands.

TGR5 has been shown to activate glucagon-like peptide 1 (GLP-1) to alleviate obesity-induced glucose intolerance [29]. However, the other TGR5 target genes involved in regulating obesity and glucose levels are elusive. The effect of TGR5 on miRNA expression has not been reported before. This study identified miR-26a as a novel target gene of TGR5. Because we have recently demonstrated a critical role of miR-26a in glucose regulation and lipid metabolism [15, 17], activation of miR-26a by TGR5 could be one of the downstream pathways by which TGR5 makes its regulation on obesity and glucose control. The potential targets of miR-26a in the macrophage include NF-B and interleukin 6 (IL-6), suggesting an anti-inflammation function of miR-26a, thereby improving insulin sensitivity and energy metabolism. Of note, miR-26a may work together with other miRNAs and pathways to achieve the pleiotropic effect of TGR5 activation. Previously, miR-26a is shown to be co-transcribed with its host genes [30,31]. A similar phenomenon is reported by another study, in which miR-26b is co-transcribed with its host gene *CTDSP1* [32]. However, this type of regulation is not conserved under all conditions. Our current study suggests a new mechanism by which TGR5 activation can directly regulate miR-26a transcription independent of its host genes. We had also mapped the TGR5-responsible DNA element to the proximal region of miR-26a promoter. The detailed regulation and identification of transcription factors that activate miR-26a transcription downstream of TGR5 will be our future interest.

In summary, the present work demonstrates that membrane-bound BA receptor TGR5 activates miR-26a expression through a JNK-dependent pathway. Our results thus provide a novel link between TGR5 and miRNA expression. These findings also suggest that activation of TGR5 by small molecules could be a novel approach to modulate the expression of miR-26a for the treatment of metabolic diseases.

## Supporting Information

S1 Fig TGR5 responsible element is proximal to miR-26a promoter. (A) Genomic location of miR-26a family members (miR-26a-1, miR-26a-2 and miR-26b). The promoters of miR-26a family members are shown. (B) RT-PCR strategy for detection of pri- and mature miR-26a.
(TIF)

S2 Fig White adipose weight of either WT or TGR5^-/-^ mice. At the end of the feeding, mice were euthanized and the white adipose tissues from either WT or TGR5-/- mice were weighted and compared.
(TIF)

S3 Fig H&E staining of mouse livers. At the end of the feeding, mice were euthanized and the liver tissue sections from either WT or TGR5^-/-^ mice were subjected to H&E staining.
(TIF)

S4 Fig Induction of FXR target gene expression in liver and intestine by 1% CA diet and GW4064. WT mice were fed with regular chow diet (Re) or 1% CA for 3 days. WT mice were orally gavaged with vehicle (Veh) or GW4064 (GW, 50 mg/kg) once every day for 3 days. MiR26a expression in the liver was determined by QRT-PCR.
(TIF)

S5 Fig DY284, a recent identified TGR5 ligand, induces miR-26a expression in mice. Wild type (WT) and TGR5^-/-^ (TGR5) mice were i.p injected with DY284 (30 mg/kg). Two days later, livers RNAs were prepared and used for measuring miR-26a levels by QRT-PCR.
(TIF)

S6 Fig Expression of miR-26a in OA-treated melanoma cell lines. SK-MEL-28 and A375 melanoma cell lines were treated with OA (10 M) for 24h. The expression of miR-26a was measured by QRT-PCR.
(TIF)
