# Supplementary figures and images for: Identification of miR-26a as a Target Gene of Bile Acid Receptor GPBAR-1/TGR5
Source: PLoS One. 2015 Jun 24;10(6):e0131294. doi: 10.1371/journal.pone.0131294 (PMC4481113; doi:10.1371/journal.pone.0131294)

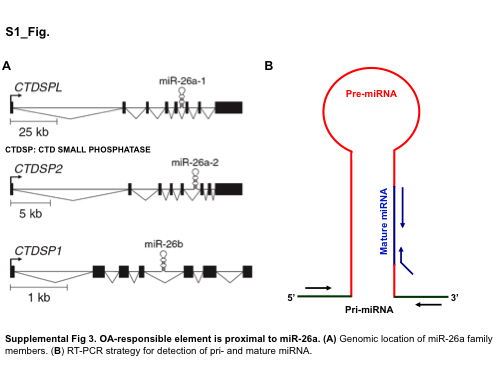

Supplement: S1 Fig — (A) Genomic location of miR-26a family members (miR-26a-1, miR-26a-2 and miR-26b). The promoters of miR-26a family members are shown. (B) RT-PCR strategy for detection of pri- and mature miR-26a. (TIF) [file pone.0131294.s001.tif]

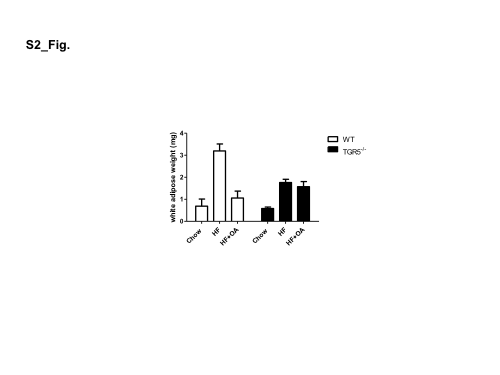

Supplement: S2 Fig — At the end of the feeding, mice were euthanized and the white adipose tissues from either WT or TGR5-/- mice were weighted and compared. (TIF) [file pone.0131294.s002.tif]

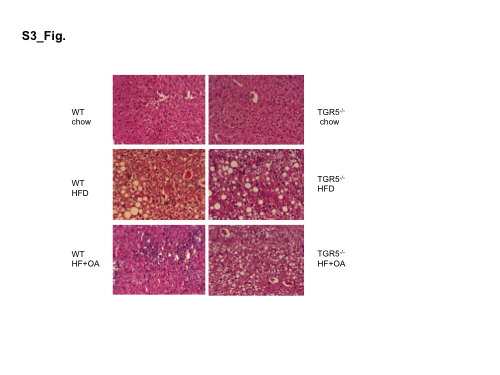

Supplement: S3 Fig — At the end of the feeding, mice were euthanized and the liver tissue sections from either WT or TGR5-/- mice were subjected to H&E staining. (TIF) [file pone.0131294.s003.tif]

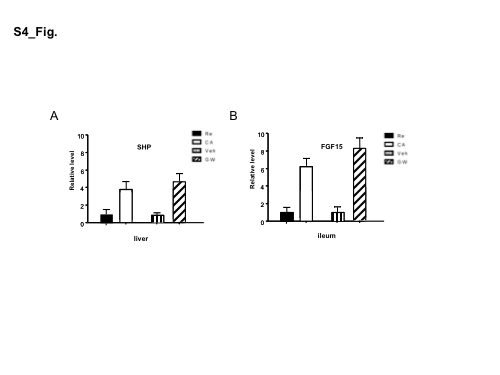

Supplement: S4 Fig — WT mice were fed with regular chow diet (Re) or 1% CA for 3 days. WT mice were orally gavaged with vehicle (Veh) or GW4064 (GW, 50 mg/kg) once every day for 3 days. MiR26a expression in the liver was determined by QRT-PCR. (TIF) [file pone.0131294.s004.tif]

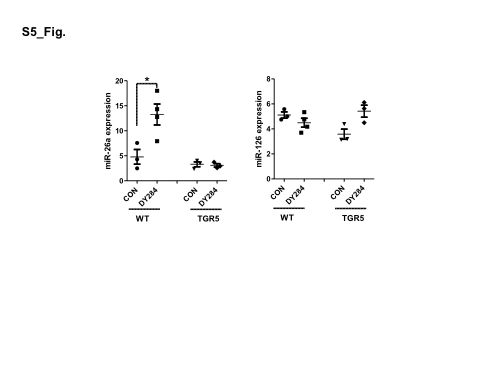

Supplement: S5 Fig — Wild type (WT) and TGR5-/- (TGR5) mice were i.p injected with DY284 (30 mg/kg). Two days later, livers RNAs were prepared and used for measuring miR-26a levels by QRT-PCR. (TIF) [file pone.0131294.s005.tif]

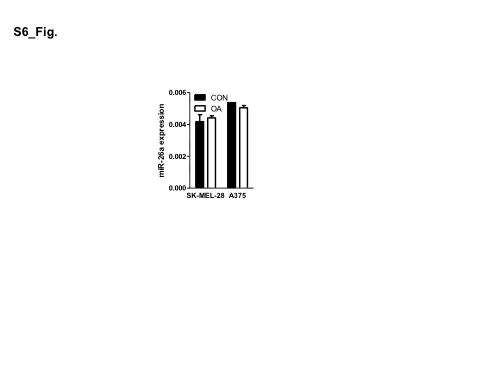

Supplement: S6 Fig — SK-MEL-28 and A375 melanoma cell lines were treated with OA (10 M) for 24h. The expression of miR-26a was measured by QRT-PCR. (TIF) [file pone.0131294.s006.tif]
